# Supplementary material for: Identifying signatures of past and present cryovolcanism on Europa
Source: Nat Commun. 2025 Feb 22;16:1886. doi: 10.1038/s41467-025-57070-8 (PMC11846997; doi:10.1038/s41467-025-57070-8)
Supplement: Supplementary file 1 — Supplementary Information [file 41467_2025_57070_MOESM1_ESM.pdf]

Identifying signatures of past and present cryovolcanism on Europa – Supplementary information

Elodie Lesage[1*], Samuel M. Howell[1], Marc Neveu[2,3], Julia W. Miller[1,4], Mariam Naseem[5], Mohit Melwani Daswani[1], Justine Villette[6] and Steven D. Vance[1]

[1*]Jet Propulsion Laboratory, California Institute of Technology, Pasadena, CA, USA.

[2]Department of Astronomy, University of Maryland, College Park, MD, USA.

[3]Planetary Environments Laboratory, NASA Goddard Space Flight Center, Greenbelt, MD, USA.

[4]Department of Earth, Environmental, and Planetary Sciences, Brown University, Providence, RI, USA.

[5]Department of Geology, University of Maryland, College Park, MD, USA.

[6]Nantes Université, Univ Angers, Le Mans Université, CNRS, Laboratoire de Planétologie et Géosciences, LPG UMR 6112, 44000 Nantes, France.

*Corresponding author(s). E-mail(s): elodie.lesage@jpl.nasa.gov;

Supplementary technical description of the CRYOLAVASAURUS simulation

This Supplemental Model Description details the structure and implementation of the CRYOLAVASAURUS simulation [1] describing the physico-thermal evolution of cryovolcanic liquid brine reservoirs in the icy worlds of the outer solar system. CRYOLAVASAURUS is an affectionate h omophone for the Cryolophosaurus, discovered in the Transantarctic Mountains in 1990.

CRYOLAVASAURUS is free and publicly available at https://github.com/ElodieLesage/Cryolavasaurus. The version used to obtain the results presented in this study, as well as all the input, output and data files are archived at https://doi.org/10.5281/zenodo.14768339.

This supplement provides technical details on key aspects of the working structure of the software package. The CRYOLAVASAURUS simulation consists of several key individual models and calculations. At a high level, a conservative spherically symmetric 1-Dimensional (1D) finite-difference structure is created on a staggered Eulerian grid. At the appropriate nodes and elements, temperature, pressure, physical and thermal material properties, phases, compositions, etc. are stored. These values are then accessed by several constitutive components of the simulation.

Section 1 describes the largest and most computationally taxing component of the simulation: a solver that treats the conservation of heat energy, appropriately considering the temperature-dependent thermal conductivity and the idiosyncrasies of the solver implementation on the spherically symmetric grid. In the following, we refer to this as the "thermal solver". This solver is active over the whole domain, where its primary purpose is to solve for the diffusion and advection of heat energy. At the surface, in the reservoir, in the ocean, and at key interfaces, the temperature solution itself is conservatively overridden by other components of CRYOLAVASAURUS that take as input the computed energy fluxes. The thermal solver considers the temperature dependence of thermal expansivity, density, thermal conductivity, and specific heat capacity.

Section 2 describes a separate solver that maintains a self-consistent surface temperature (and therefore observable temperature anomaly), interfacing with the thermal solver as a dynamic surface heat flux boundary condition. This heat flux balance takes into account the various sources and sinks of thermal energy at the surface, and derives a heat-flux anomaly driven by a mismatch of surface temperature calculated in the previous timestep with the surface temperature that satisfies the heat flux balance. This anomaly is then propagated as a negative surface energy flux in the thermal solver, which elicits the self-consistent evolution of surface temperature.

Section 3 describes a subroutine responsible for invoking compositional lookup tables. These lookup tables are the results of previous modeling efforts that analyzed the thermal and compositional evolution of plausible Europa ocean compositions [2] as a function of decreasing temperature below 273 K [3], using PHREEQC [4]. These lookup tables are then used algorithmically to prescribe the partitioning of changes in total enthalpy produced by the modified thermal solver across changes in temperature, phase, and composition. Updates are then conservatively applied to the values stored on the model grid.

Section 4 describes a separate solver that maintains the conservation of enthalpy associated with phase and compositional changes, interacting with the thermal solver as a dynamic internal boundary condition. The enthalpies of fusion and formation of the various compositional constituents are invoked

to understand the sources and sinks of energy within the model associated with melting, freezing, and fractionation. Such changes are then propagated back into the thermal solver as energy flux corrections, and the temperature solutions within the ocean, reservoir, and at the interfaces are prescribed on the grid self-consistently.

Section 5 describes a subroutine responsible for the accumulation and relaxation of reservoir stresses in response to freezing. Volume expansion during freezing conserves enthalpy by inducing an increase in pressure. Using fundamental descriptions of compressibility and viscoelasticity, we predict the occurrence of reservoir eruptions when internal pressurization overcomes the mechanical resistance of the overlying ice. This subroutine tracks its own results separately from the main simulation and feeds back into the simulation by providing a source and sink of enthalpy stored mechanically as reservoir pressure.

1 Conservation of heat

We describe here the numerical procedure used to model heat transfer across a spherical body. To model heat conduction and diffusion, we apply the conservative finite differences method forward in time in 1D spherical coordinates as described in [5]. Heat diffusion is governed by the equation:

$$\rho c_p \frac{\partial T}{\partial t} = -\frac{\partial q}{\partial r} \tag{1}$$

$$= \frac{1}{r^2}\frac{\partial}{\partial r}\left(kr^2\frac{\partial T}{\partial r}\right), \tag{2}$$

with ρ the solid density (kg m^{-3}), c_p the solid specific heat capacity (J kg^{-1} K^{-1}), T the temperature field (K), r the radial distance from the sphere center (m), q the heat flux (J s^{-1} m^{-2}) and k the thermal conductivity (J s^{-1} m $^{-1}$ K^{-1}).

We discretize Eq. (1) using an Eulerian staggered grid, with the staggered nodes spaced to correctly conserve heat flow on a spherical grid, maintaining numerical stability. The nodes are assigned radially outward. We use the subscript i to denote the node indices in the following equations. In the following equations, we use the subscripts A and B to denote the staggered elements located, respectively, inward and outward of node i. Using this notation, the discretization of Eq. (1) gives:

$$\rho_i c_{p,i}\frac{T_i^{n+1} - T_i^n}{\Delta t} = -\frac{1}{r_i^2}\frac{r_B^2 q_B^n - r_A^2 q_A^n}{dr}. \tag{3}$$

where the superscripts n and $n+1$ denote quantities at the current and the next time step, respectively. The position of the staggered elements r_A and r_B

are chosen such that:

$$r_A = \sqrt{r_{i-1} r_i}, \tag{4}$$
$$r_B = \sqrt{r_i r_{i+1}}. \tag{5}$$

Heat flux is evaluated at the staggered nodes to avoid jumps in the energy fluxes due to variation of the physical parameters at the nodes, providing a conservative description of the heat flux [5]. Heat fluxes q_A and q_B are given by:

$$q_A = -Nu\, k_A \frac{T_i - T_{i-1}}{dr}, \tag{6}$$
$$q_B = -Nu\, k_B \frac{T_{i+1} - T_i}{dr}. \tag{7}$$

where Nu is the Nusselt number, which is used to account for convection, and k_A and k_B are the thermal conductivity coefficient averaged across the staggered elements. The values at A and B and are calculated conservatively using a harmonic mean:

$$k_A = \frac{k_{i-1} k_i (r_i - r_{i-1})}{k_i (r_A - r_{i-1}) + k_{i-1}(r_i - r_A)}, \tag{8}$$
$$k_B = \frac{k_i k_{i+1} (r_{i+1} - r_i)}{k_{i+1} (r_B - r_i) + k_i (r_{i+1} - r_B)}. \tag{9}$$

At the solid-liquid interfaces (ice-ocean, ice-reservoir bottom, ice-reservoir top), the staggered elements bounding the interface node are partially molten. The interface location within the nodes is tracked, and the thermal and physical properties at the node are a volume- or mass-average of those within the staggered elements, as appropriate. To consider the effective infinite thermal conductivity of a vigorously convecting ocean or reservoir, and avoid a diverging thermal conductivity when the node is almost fully liquid, we use a harmonic mean of the thermal conductivity across the full volume elements immediately below and immediately above the interface. Let the node *int* be the interface node, we obtain an effective thermal conductivity at this node:

$$k_{eff} = \left(\frac{\frac{dV_{int-1}\phi_{int-1}}{k_{int-1}} + \frac{dV_{int}\phi_{int}}{k_{int}} + \frac{dV_{int+1}\phi_{int+1}}{k_{int+1}}}{dV_{i-1} + dV_i + dV_{i+1}} \right)^{-1} \tag{10}$$

where dV_i is the volume of the spherical element i situated just outward of the node i, and ϕ_i is the volume fraction of liquid in the node (ϕ=1 if the node is fully liquid, ϕ=0 if the node is fully solid). We then use this averaging to prescribe $k_A = k_B = k_{eff}$, and issue values for the three nodes $int - 1$, int

and $int + 1$. This lowers the effective spatial resolution of conduction at the interface, but preserves numerical stability. Because the interface is carefully and conservatively tracked by the phase and compositional enthalpy change solver, there is no measurable degradation of solution accuracy.

Finally, the temperature change at each node between two time steps is calculated as:

$$T_i^{n+1} - T_i^n = -\frac{dt}{\rho_i c_{p_i} r_i^2 dr}\left(k_A r_A^2 \frac{T_{i+1} - T_i}{dr} - k_B r_B^2 \frac{T_i - T_{i-1}}{dr}\right). \tag{11}$$

To ensure that the model is energetically conservative, we use the Courant-Friedrichs-Lewy (CFL) criterion: the time step used should always be less than the time it takes for the heat to travel through one full element [5]. In our model, we must explicitly consider remaining conservative against conductive heat transport (in the ice shell) and radiative emission (at the surface). At the surface, heat might be radiated to space at an effective thermal diffusivity far in excess of the icy values, resulting in effective heat transport timescales much shorter than conduction. Although this is also true within the ocean and reservoir, where the effective thermal conductivity of the liquids is treated as infinite, the phase and compositional enthalpy conservation solver overprints the thermal solution in these regions, maintaining numerical stability and conserving energy. For this reason, we estimate the heat transport time scales associated with both conduction and radiation, choosing the shortest relevant timescale. The conductive and radiative timescales are respectively given by:

$$dt_{cond} = \frac{dr^2}{\max(\kappa Nu)} \tag{12}$$

and

$$dt_{emis} = \frac{dr \rho_{surf} cp_{surf} T_{surf}}{q_{sol}}, \tag{13}$$

where dr is the radial extend of a volume element, $\kappa = \frac{k}{\rho Cp}$ is the ice thermal diffusivity (m s^{-1}), Nu is the Nusselt number, q_{sol} is the average solar insolation flux (W m^2) and ρ_{surf}, cp_{surf} and T_{surf} are respectively the density, specific heat capacity, and temperature at the surface node. Finally, we take:

$$dt = \mathrm{CFL} \min(dt_{cond}, dt_{emis}), \tag{14}$$

where CFL is the Courant–Friedrichs–Lewy coefficient, here implemented as $\frac{1}{10}$ to maintain numerical stabillity.

2 Surface heat flux balance

At the surface, the temperature evolves self-consistently by balancing incoming diurnally averaged equatorial insolation, $\dot{q}_{sol}$, and geologic heat flow

through the surface, $\dot{q}_{geo}$, with heat losses due to blackbody radiation, $\dot{q}_{bb}$, and sublimation to maintain a rarefied atmosphere, $\dot{q}_{sub}$:

$$\dot{q}_{sol} + \dot{q}_{geo} = \dot{q}_{bb} + \dot{q}_{sub}. \tag{15}$$

The diurnally averaged equatorial insolation is prescribed following Ojakangas and Stevenson (1989) [6]:

$$\dot{q}_{sol} = (1 - A) \left(\frac{\dot{E}_{sol}}{4\pi R_J^2} \frac{\sqrt{\delta^2 + \phi^2}}{\pi \sqrt{2}} \right), \tag{16}$$

where A is the effective surface-averaged bolometric albedo, 0.55, $\dot{E}_{sol}$ is the total solar power of the Sun, 3.8×10^{26} W, R_J is the average orbital distance of Jupiter from the Sun, 7.78×10^{11} km, δ is the obliquity (axial tilt) of Europa in radians, 5.24×10^{-2}, and ϕ is the co-latitude of interest in radians, 0 in the case of an equatorial average [6].

The geologic heat flux is calculated by the full conservation of enthalpy solver, which internally tracks heat conduction out of the boundary element in response to the cumulative generation of tidal and radiogenic heat, and exchange and transport of heat within the interior.

The heat flux leaving Europa as it radiates energy to space is determined simply by the Stefan-Boltzmann law:

$$\dot{q}_{bb} = \epsilon \varsigma T_s^4, \tag{17}$$

where ϵ is the effective surface-averaged emissivity, 0.9, ς is the Stefan-Boltzmann constant, 5.67×10^{-8} W m^{-2} K^{-4}, and T_s is the surface temperature.

Heat is also effectively partitioned into the specific enthalpy of water vapor formation at the surface to maintain a partial pressure of water vapor in equilibrium with the sublimation temperature of ice to form a rarefied atmosphere. Acting as a free kinetic gas, water molecules lose the stored enthalpy of sublimation through radiative cooling and inelastic collisions. Sublimation losses are calculated accordingly [7, 8]:

$$\dot{q}_{sub} = L_{sub} p_{H_2O} \sqrt{\frac{m_{H_2O}}{2\pi R T_s}}, \tag{18}$$

where L_{sub} is the latent heat of sublimation calculated as a function of T_s [8], p_{H_2O} is the partial pressure of water vapor calculated as a function of T_s [7], m_{H_2O} is the molecular weight of water, 18.01528×10^{-3} kg mol^{-1}, and R is the ideal gas constant, 8.314 J mol^{-1} K^{-1}.

At the base of the ice shell, the ocean is considered as a fixed-temperature source of thermal and phase energy with an effectively infinite thermal conductivity, simulating a vigorously convecting ocean (relative to the ice shell).

We calculate the initial surface temperature using the heat flux balance from Eq. 15. We treat the anomalous heat flux as an unknown to allow the

Table 1 Reference of temperature-dependent composition data sheets used in this study and the input oceanic composition they correspond to. Ocean compositions were obtained by [2] and the temperature-dependent composition data was obtained by [3].

Model	Sheet name	Sheet number
CM carbonaceous chondrites	Eq EM-CM carb	1
CI carbonaceous chondrites	Eq EM-CI carb	3
Chondrite mix and cometary material	Eq MC-Scale carb	5
Pure water	Pure Water	17

model to be self-consistent. To do so, we use an iterative process to solve for Eq. 15: we propose a first guess $T_{surf,0}$ for the surface temperature using $q_{anom} = 0$ and then calculate the associated temperature gradient in the ice shell:

$$T(z) = T_{surf,0}\left(\frac{T_{ocean}}{T_{surf,0}}\right)^{z/H_0}, \tag{19}$$

where T_{ocean} is temperature at the bottom of the ice shell and H_0 is the initial ice shell thickness. We estimate the surface heat flux associated with this thermal gradient:

$$q_{surf} = \left(k_{surf}(T_{ocean} - T_{surf,0})\right)(1/H_0 + 1/R); \tag{20}$$

where k_{surf} is the mean thermal conductivity of a conductive spherical shell spanning the ocean to the surface. Finally, we use q_{surf} as an estimate for q_{anom} and iterate over $T(z)$ and q_{anom} until they converge. This ensures that the initial surface temperature is consistent with the energy balance. To account for convective influence on ice shell thickness, estimation of the conductive temperature profile using a linear scaling, and equilibrium of the phase of the ice shell and surroundings, we allow the thermal structure to converge over 5 Myr before emplacing the reservoir.

3 Composition evolution data

The Microsoft Excel file containing the three composition data spreadsheets used in this study can be found at https://github.com/ElodieLesage/Cryolavasaurus/blob/main/CompData.xlsx [1]. In Table 3, we list the data sheets that we used to obtain the presented results. We used three different input compositions, each corresponding to a possible injected oceanic composition. The ocean compositions depend on the formation scenario considered for Europa and have been obtained by [2]. The evolution of these compositions during freezing has been modeled by [3] using PHREEQC [4] and results in the temperature-dependent concentrations we used and which are summarized in Table 3.

4 Phase and compositional enthalpy conservation

Tracking the energy exchanged at the top and bottom boundaries of the reservoir is key to relating the chemical and thermal paths of the reservoir. It allows us to trace the amount of frozen cryomagma and both phase compositions as a function of time.

For each temperature point of the PHREEQC simulations, we know the change in ice and magma somposition, the change in frozen fraction of the cryomagma, and change in temperature that corresponds to the new melting temperature. We can thus calculate the partitioning of energy across both phase change and cooling at each PHREEQC data point:

$$\delta E_{freeze} = dV_{liq}\,\rho_{liq}\,L, \tag{21}$$

$$\delta E_{cool} = V_{liq}\,dT\,\rho_{liq}\,c_p. \tag{22}$$

with V_{liq} the liquid volume, dT the temperature change in the liquid phase, ρ_{liq} the liquid density, c_p the liquid heat capacity and L the water latent heat of fusion. From the reservoir point of view, a total amount of energy $\Delta E = \delta E_{freeze} + \delta E_{cool}$ leaves the reservoir between each step. Knowing that the total amount of energy available in the reservoir at the simulation start is $E_{init} = \rho_{liq,\,init} V_{init}(T_{init}\,c_p + L)$, we can calculate the ratio

$$f_{E,\,PH} = \frac{E_{init} - \Delta E}{E_{init}} \tag{23}$$

that indicates the amount of energy remaining in the reservoir –where the subscript "PH" stands for "PHREEQC". Each value of $f_{E,\,PH}$ is then associated with a pair of values $(\delta E_{freeze}, \delta E_{cool})$. The phase and compositional enthalpy solver therefore tracks the flux of energy across each spherical volume element associated with those processes.

The thermal solver also tracks the energy exchange through the reservoir walls using Eq. (6). At each time step, we calculate the ratio of energy $f_{E,TS}$ remaining in the reservoir to the initial reservoir energy–where the subscript "TS" stands for the "thermal solver". Relating this energy flux to that calculated from the PHREEQC data, $f_{E,\,PH}$, we create a system by which the thermal solver can quantify the energy extracted each timestep from the phase interfaces and similarly accept modifications of the energy flux that compensate for phase and compositional changes.

At each time step, we compute the value of $f_{E,\,TS}$ on a spline interpolation of $f_{E,\,PH}$ and extract the associated energy partitioned across the phase change (δE_{freeze}) and the temperature change (δE_{cool}). Within the reservoir, we then calculate the amount of frozen cryomagma and the liquid temperature in the reservoir at each time step. Within this solver, we additionally leverage the $f_{E,\,PH}$ energy path to track the frozen and liquid composition at each time step.

5 Pressurization and eruption

The freezing-induced overpressure ΔP in a reservoir is calculated as:

$$\Delta P = -\frac{1}{\chi} \ln\left(\frac{V_f}{V_i}\right), \tag{24}$$

with χ the liquid water compressibility, V_f the liquid water volume compressed by freezing and V_i the volume of liquid water if it could occupy an ideal space without being compressed. If the inner overpressure exceeds a critical value ΔP_c, the reservoir wall breaks and an eruption is triggered. We calculate ΔP_c using the ice tensile strength σ at the far-field temperature:

$$\Delta P_c = 2(\sigma + \rho g H), \tag{25}$$

with $\rho = 1000$ kg m^3 the water ice density, g = 1.315 m s^{-2} Europa's gravity and H the reservoir roof depth. σ was determined experimentally at Europa-like conditions and temperatures by [9].

As demonstrated in [10], the viscous dissipation of elastically stored energy (that is, the relaxation of accumulated stresses by viscous flow) limits the ability for reservoirs to trigger eruptions by reducing the inner pressure. We account for viscoelastic relaxation of the reservoir wall when calculating the inner pressure at each time step. For a Maxwell viscoelastic material, the strain ϵ resulting from a stress σ is described by:

$$\frac{d\varepsilon}{dt} = \frac{1}{E}\frac{d\sigma}{dt} + \frac{\sigma}{\eta}, \tag{26}$$

where E is the elastic modulus (Young's modulus) and η is the temperature-dependent viscosity [11]:

$$\eta = \eta_0 \exp\left\{25.2\left(273/T - 1\right)\right\}. \tag{27}$$

with η_0 the viscosity that maximizes tidal dissipation at the melting temperature [12]. After strain has accumulated ($d\varepsilon/dt = 0$), we can integrate Eq. (26) to obtain the stress $\Delta\sigma$ propagated over a time step Δt:

$$\Delta\sigma = \sigma e^{-\Delta t/\tau_M}. \tag{28}$$

where $\tau_M = \eta/E$ is the Maxwell time scale. At each time step, n, the pressure in the reservoir is the sum of the pressure ΔP^{n+1} generated by freezing over the time step and the unrelaxed/unrelieved pressure propagated from the previous time step using 28, accounting for the interim relaxation:

$$P^{n+1} = \left(\Delta P^{n-1} + P^{n-1}\right) e^{-\Delta t E/\eta}. \tag{29}$$

References

[1] Lesage, E., Howell, S. M. & Miller, J. W. CRYOLAVASURUS. https://github.com/ElodieLesage/Cryolavasaurus/releases/tag/v1 (2025). URL https://doi.org/10.5281/zenodo.14768339.

[2] Melwani Daswani, M., Vance, S. D., Mayne, M. J. & Glein, C. R. A metamorphic origin for Europa's ocean. *Geophysical Research Letters* **48** (18), e2021GL094143 (2021). https://doi.org/https://doi.org/10.1029/2021GL094143 .

[3] Naseem, M. *et al.* Salt distribution from freezing intrusions in ice shells on Ocean Worlds. *The Planetary Science Journal* **4**, 181 (2023). https://doi.org/10.3847/PSJ/ace5a2 .

[4] Parkhurst, D. L. & Appelo, C. Description of input and examples for PHREEQC version 3—a computer program for speciation, batch-reaction, one-dimensional transport, and inverse geochemical calculations. *US Geological Survey Techniques and Methods* **6** (A43), 497 (2013). URL https://pubs.usgs.gov/tm/06/a43/ .

[5] Gerya, T. *Introduction to Numerical Geodynamic Modelling* (CAMBRIDGE, 2019). URL https://www.ebook.de/de/product/34700198/taras_gerya_introduction_to_numerical_geodynamic_modelling.html.

[6] Ojakangas, G. & Stevenson, D. J. Thermal state of an ice shell on Europa. *Icarus* **81** (2), 220–241 (1989). https://doi.org/10.1016/0019-1035(89)90052-3 .

[7] Marti, J. & Mauersberger, K. A survey and new measurements of ice vapor pressure at temperatures between 170 and 250 K. *Geophysical Research Letters* **20** (5), 363–366 (1993). https://doi.org/https://doi.org/10.1029/93GL00105 .

[8] Yau, M. K. & Rogers, R. R. *A short course in cloud physics* (Elsevier, 1996).

[9] Litwin, K. L., Zygielbaum, B. R., Polito, P. J., Sklar, L. S. & Collins, G. C. Influence of temperature, composition, and grain size on the tensile failure of water ice: Implications for erosion on Titan. *Journal of Geophysical Research* **117** (E08013) (2012). https://doi.org/10.1029/2012je004101 .

[10] Lesage, E., Massol, H., Howell, S. M. & Schmidt, F. Simulation of freezing cryomagma reservoirs in viscoelastic ice shells. *The Planetary Science Journal* **3** (7), 170 (2022). https://doi.org/10.3847/PSJ/ac75bf .

[11] Goldsby, D. & Kohlstedt, D. L. Superplastic deformation of ice: Experimental observations. *Journal of Geophysical Research: Solid Earth* **106** (B6) .

[12] Howell, S. M. The likely thickness of Europa's icy shell. *The Planetary Science Journal* **2** (4), 129 (2021). https://doi.org/10.3847/psj/abfe10 .
